# Evaluation of the Effects of 1,25VitD3 on Inflammatory Responses and IL-25 Expression

**DOI:** 10.3389/fgene.2021.779494

**Published:** 2021-12-06

**Authors:** Nana Li, Nafiseh Saghafi, Zahra Ghaneifar, Seyed Abdorahim Rezaee, Houshang Rafatpanah, Elham Abdollahi

**Affiliations:** ^1^ Department of Obstetrics, Jinan Maternal and Child Care Health Hospital Affiliated to Shandong First Medical University, Jinan, China; ^2^ Department of Gynecology, Woman Health Research Center, Mashhad University of Medical Sciences, Mashhad, Iran; ^3^ Department of Nutrition, School of Medicine, Mashhad University of Medical Sciences, Mashhad, Iran; ^4^ Department of Immunology and Allergy, School of Medicine, Mashhad University of Medical Sciences, Mashhad, Iran; ^5^ Research Center for HIV/AIDS, HTLV and Viral Hepatitis, Iranian Academic Center for Education, Culture, and Research (ACECR), Mashhad Branch, Mashhad, Iran; ^6^ Inflammation and Inflammatory Diseases Research Center, School of Medicine, Mashhad University of Medical Sciences, Mashhad, Iran

**Keywords:** URSA, VitD3, Th17 cells, IL-25, inflammation

## Abstract

VitD3 may contribute to a successful pregnancy through modulation of immune responses, so VitD3 deficiency may have a role in the immunopathogenesis of unexplained recurrent spontaneous abortion (URSA). However, the mechanisms of immunomodulatory actions of VitD3 in decreasing the risk of recurrent spontaneous abortion have not been understood well.

**Objective:** The purpose of this research was to investigate the influence of 1,25VitD3 on IL-25 and related cytokines of Th17 cells including IL-17A, IL-6, and IL-23 in peripheral blood mononuclear cells of healthy women as a control group and women with unexplained recurrent spontaneous abortion.

**Method:** Isolation of peripheral blood mononuclear cells (PBMCs) was performed from peripheral blood of the subjects of the studied groups (20 women with URSA as a case group, and 20 control women). The effects of 1,25VitD3 (50 nM, for 24 h) on the studied parameters were evaluated and were compared to the positive and negative controls *in vitro*. Flow cytometry analysis was used to determine the percentages of regulatory T cells and Th17 cells. For gene expression measurement and cytokines assay, real-time PCR and ELISA were carried out.

**Results:** The proportion of Th17 cells in women with URSA was considerably higher than in the control group. IL-25 mRNA and protein levels in cultured PBMCs from women with URSA were lower than the controls. 1,25VitD3 increased IL-25 expressions at both the protein and mRNA levels in PBMCs from women with URSA relative to the control group. Additionally, 1,25VitD3 treatment not only significantly decreased the percentage of Th17 cells frequency but also reduced expressions of IL-6, IL-17A, and IL-23 in PBMCs from women with URSA.

**Conclusion:** 1,25VitD3 may diminish inflammatory responses cells *via* downregulation of IL-25 expression. It could be an interesting subject for future researches in the field of the immunopathology of URSA to identify molecular pathways in URSA treatment.

## Introduction

One of the most serious pregnancy complications is unexplained recurrent spontaneous abortion (URSA), which refers to three or more consecutive pregnancy losses before 20 completed gestation weeks (Dimitriadis et al., 2020). URSA is usually 50% without any recognizable endocrine, genetic, infectious, and anatomical factor that affects 1–5% of fertile women (Dimitriadis et al., 2020; Giannubilo et al., 2012). Idiopathic abortions are caused by disrupted immune responses, according to new evidence (Guerin et al., 2009; Abdollahi et al., 2020a). Interleukin-25 (IL-17E) is a cytokine in the IL-17 family with a sequence that is 16–20% identical to that of IL-17A (Pan et al., 2001; Kolls and Lindén, 2004; Iwakura et al., 2011). In terms of structure and biological function, IL-25 is distinct from other members of the IL-17 family (Lee et al., 2001; Pan et al., 2001; Kolls and Lindén, 2004; Iwakura et al., 2011; Mantani et al., 2015). IL-25 is released by activated Th2 cells, bone marrow-derived mast cells, vascular endothelial cells, alveolar macrophages, basophils, and eosinophils (Fort et al., 2001; Pan et al., 2001; Kim et al., 2002; Wang et al., 2007; Divekar and Kita, 2015). Peripheral blood mononuclear cells (PBMCs), especially CD4^+^ T cells, are the main sources of IL-25 in the bloodstream (Licona-Limón et al., 2013; Fahy, 2015; Barati et al., 2020). CD4^+^T cells, which include T helper 1 (Th1), Th2, regulatory T cells (Tregs), and Th17 cells, play an important role in the maternal immune response (Caruso et al., 2009; Saito et al., 2010; Figueiredo and Schumacher, 2016; Barad et al., 2017; Cyprian et al., 2019; Pei et al., 2019; Muyayalo et al., 2020).

Th17 cells elicit inflammatory reactions by producing cytokines such as IL-22, IL-21, IL-17A, IL-17 F, and TNFα as the pro-inflammatory cytokines (Veldhoen et al., 2006; Abdollahi et al., 2015a; Abdollahi et al., 2016a; Konkel et al., 2017; Fujimoto et al., 2020).

The active form of VitD3 (1,25(OH)2D3, 1,25VitD3) is a multi-target hormone (Heyden and Wimalawansa, 2018) that has a critical role in bone health by regulating calcium and phosphate homeostasis (Eisman et al., 1979; Makishima et al., 2002; Veldurthy et al., 2016). Beyond the classical function, it was found that upon binding to vitamin D receptor, VitD3 regulates function and differentiation of different immune cells including macrophages, B cells, dendritic cells, and T cells (Umar et al., 2018; Wang et al., 2020). VitD3 has been shown to contribute to decidualization and implantation *via* the modulation of inflammatory and immune responses leading to successful pregnancy (Luk et al., 2012; Dabrowski et al., 2015; Gonçalves et al., 2018; Martens et al., 2020). In 85% of pregnant women, VitD3 deficiency is common and may be associated with an increased risk of pregnancy complications, such as preeclampsia, infertility, and abortion (Vijayendra Chary et al., 2015; Blomberg Jensen et al., 2016).

To our knowledge, no research has been carried on the evaluation of IL-25 expression and Th17 responses in PBMCs from women with URSA compared to the healthy women (as the controls) in the presence of 1,25 VitD3. In this study, we evaluated the frequency of Th17 cells, as well as the levels of IL-25, IL-17A, and IL-6 as related cytokines in PBMCs from women with URSA and controls, as well as the possible correlations.

## Materials and Methods

### Subjects

This was a case-control study that was conducted from 2019 to 2021 on 20 non-pregnant women with RSA (the case group), and 20 fertile non-pregnant women (the control group). The controls had at least one normal delivery. Women in both the case and the control groups were at reproductive age and were not pregnant as indicated by a negative result in the blood HCG test. They were with regular menstruation, a normal BMI, and without any anatomical or genetic abnormalities. The women in the case group did not have any medical problems (except for RSA) and did not take any medication. The exclusion criteria for the case group were fewer than three consecutive miscarriages, positive screening tests such as hormone tests, viral infections (HIV, HBV and, HCV), agglutination assay (TPPA) for detection of antibodies against the causative agent of syphilis, autoantibodies (anti-phospholipid antibodies, antinuclear antibodies, anti-cardiolipin antibodies, lupus anticoagulant antibodies), female and male, and karyotypes.

Inclusion criteria for the case group were normal results in the mentioned lab test panel, VitD3 deficiency (less than 20 ng/ml), as well as no consumption of vitamin D supplements in the previous 3 months.

Inclusion criteria for the controls were normal results in the routine lab test panel (as mentioned above), VitD3 deficiency, no consumption of vitamin D supplements in the previous 3 months, no history of miscarriage, and they had at least one healthy child.

Semen analysis was conducted for male partners of all studied women to ensure that sperm count, sperm shape, and movement were normal.

### Isolation of PBMCs

PBMCs isolation was carried out by Ficoll, lymphosep (Biosera, UK) from 10 ml of peripheral blood. After twice PBMC washing with PBS (phosphate-buffered saline, Sigma-Aldrich, Israel), 10^6^ cells/ml were cultured in media (RPMI-1640 with 10% heat-inactivated fetal calf serum (FCS), 100 U/ml Pen-Strep, 2 mM L-Gln). For evaluation of cell viability, Trypan Blue dye exclusion was used.

### Optimization of 1,25VitD3 Concentration

1,25VitD3 (50 nM) for 24 h was selected as the optimized concentration and time of treatment. Six-well plates were used for PBMC culture (2 × 10^5^/ml of media) with adding the different concentrations of 1,25 VitD3 [10, 30, 50, 100 nM, and 0 (control)] for 12, 24, 48 and 72 h. Optimization of the concentration of 1,25VitD3 was achieved by assessing the proportion of Tregs and Th17 cells among isolated PBMCs from four women with RSA, using flow cytometry analysis FACS Calibur (BD, USA).

### Cell Culture

PBMC (2×10^6^/subject) was seeded in each well of six well plates. For each subject (for flow cytometry and real-time PCR analyses), there were four experiments as described in the previously published study (Abdollahi et al., 2020b): 1. with 1,25VitD3 (Sigma, Israel, 50 nM for 24 h) treatment; 2. with PHA (Gibco Company, USA, 10 µM) treatment; 3. with the media only (as the baseline), 4. uncultured PBMCS.

### Flow Cytometry Detection of Th17 Cells

PBMCs (1×10^6^) were treated with 50 ng/ml PMA (eBioscience, USA) and 1 μg/ml ionomycin (eBioscience, USA) to stimulate for intracellular cytokine production (for 5 h in the presence of brefeldin A (eBioscience, USA) at 37°C and 5% CO_2_). After that, cells were stained for surface of the markers with anti-CD8 conjugated with FITC and anti-CD3 conjugated with PE-Cy5 (BD Biosciences, USA) using the required buffers. The cells were fixed/permeabilized buffer (eBioscience, USA). Isotype control or anti-IL17 (PE-conjugated) was used for intracellular staining of Th17 cells (eBiosciences, USA).

### Flow Cytometry Assessment

FACS Calibur system was used for flow cytometry assessment (1×10^5^ cells). The data were analyzed by the Cell Quest software (Becton Dickinson, USA).

### Real-Time PCR

RNA extraction kit (Invitek, Germany) was used for total RNA extraction from PBMCs according to the manufacturer’s instructions. Reverse transcriptions were performed by RevertAid™ Hprimers (Germany). Primer-BLAST was performed to verify the specificity of primers. Checking RNA quality was carried out by agarose gel (2%) electrophoresis that appeared 5.8, 18, and 28 S bands by a UV light transilluminator. The total volume of all PCR reactions was 20 μl containing 10 µl of Real-time PCR -SYBR Green Master Mix (Takara, Japan), 0.3 µl of each primer (Table 1), and 7.4 µl of RNase-free water. Rotor-Gene Q cycler (Qiagen, Germany) performed real-time PCR. The following standard PCR reaction conditions were used for all transcripts: 10 min at 95°C, 15 s at 95°C (45 cycles), 30 s at 57°C, and 1 min at 60°C.

**TABLE 1:** Primer sequences used in real-time quantitative reverse transcriptase polymerase chain reaction analysis.

Target gene	Sequence 5–3′	Purpose	Product length (bp)
β2M	5′-TTG​TCT​TTC​AGC​AAG​GAC​TGG-3′	Forward Reverse	127
	5′-CCA​CTT​AAC​TAT​CTT​GGG​CTG​TG-3′		
IL-25	5′-ACT​ACT​TCA​AGT​TCC​ACA​ACA​TGC-3′	Forward Reverse	112
	5′GAG​TGT​CCG​CTG​CTT​CTC​TG-3′		

Logarithmic dilution series of the total RNA was used to construct 10-fold dilution standard curves for IL-23, IL-6, IL-25, and IL-17A. B2M was used as the internal control gene to normalize mRNA levels between the mentioned cytokines.

### ELISA

Serum VitD3 levels and sex hormones (FSH, LH, Estradiol, Progesterone, and Prolactin) of all subjects were measured by ELISA (25-Hydroxy vitamin kit, EuroImmune, Germany) following the manufacturer’s protocol.

To measure the levels of cytokines, PBMCs (1 × 10^6^ cells/ml) were cultured in RPMI media (which was described previously) with 50 nM 1,25VitD3 or absence of that. Cell culture supernatants were collected after 72 h and were assayed for concentrations of soluble IL-10, IL-25, IL6, IL-17A, and IL-23 by linked immunosorbent assay using ELISA kits (Biolegend, USA). All samples were run in duplicate. The sensitivity of each assay was as follows: 0.8 pg/ml (IL-17A), 1.6 pg/ml (IL-6), 3.5 pg/ml (IL-25), and 2.45 pg/ml for IL-23. Coefficients of variation (CV) were <10% and <5% for inter-assay and intra-assay, respectively.

### Analysis of Statistics

SPSS 16.0 software was used for statistical analysis. Mean data were compared by ANOVA and parametric T-test. P-values of less than 0.05 were regarded as significant*.* For analyzing all of the inter-group comparisons (the comparison of the studied parameters between the case and the control groups), and for accurate normalization, 1,25VitD3/untreated relative gene expression of between two groups was compared. The data is presented as mean ± standard error (SE).

## Results

There was no significant difference in mean age between the control group [27.23 ± 3.5 years (range 24–31 years)] and women with RSA [29.72 ± 2.9 years (range 26–32 years)] (*p* > 0.05). No significant variations were found in the serum level of sexual hormones between the case and the control groups (Table 2 **)**. VitD3 serum levels were statically similar between the two groups (7.8 ng/ml ± 1.2 versus 6.9 ng/ml ± 1.0; *p* > 0.05).

**TABLE 2:** Sex hormone levels of the case and control groups.

Sex hormone	Case group	Control group	P value
	N = 20	N = 20	
FSH(mIU/ml)	4.6 ± 3.8	5.4 ± 3.66	0.36
LH (mIU/ml)	12.8 ± 17.8	12.1 ± 16.4	0.89
Prolactin (ng/ml)	21.4 ± 22.8	16.0 ± 20.6	0.24
Progesterone (ng/ml)	4.5 ± 6.8	5.2 ± 5.4	0.72
Estradiol (pg/ml)	15.1 ± 19.0	14.7 ± 19.3	0.73

1,25VitD3 increased IL-25 expressions at both protein and mRNA levels in women with URSA relative to the control group.

**FIGURE 1:**
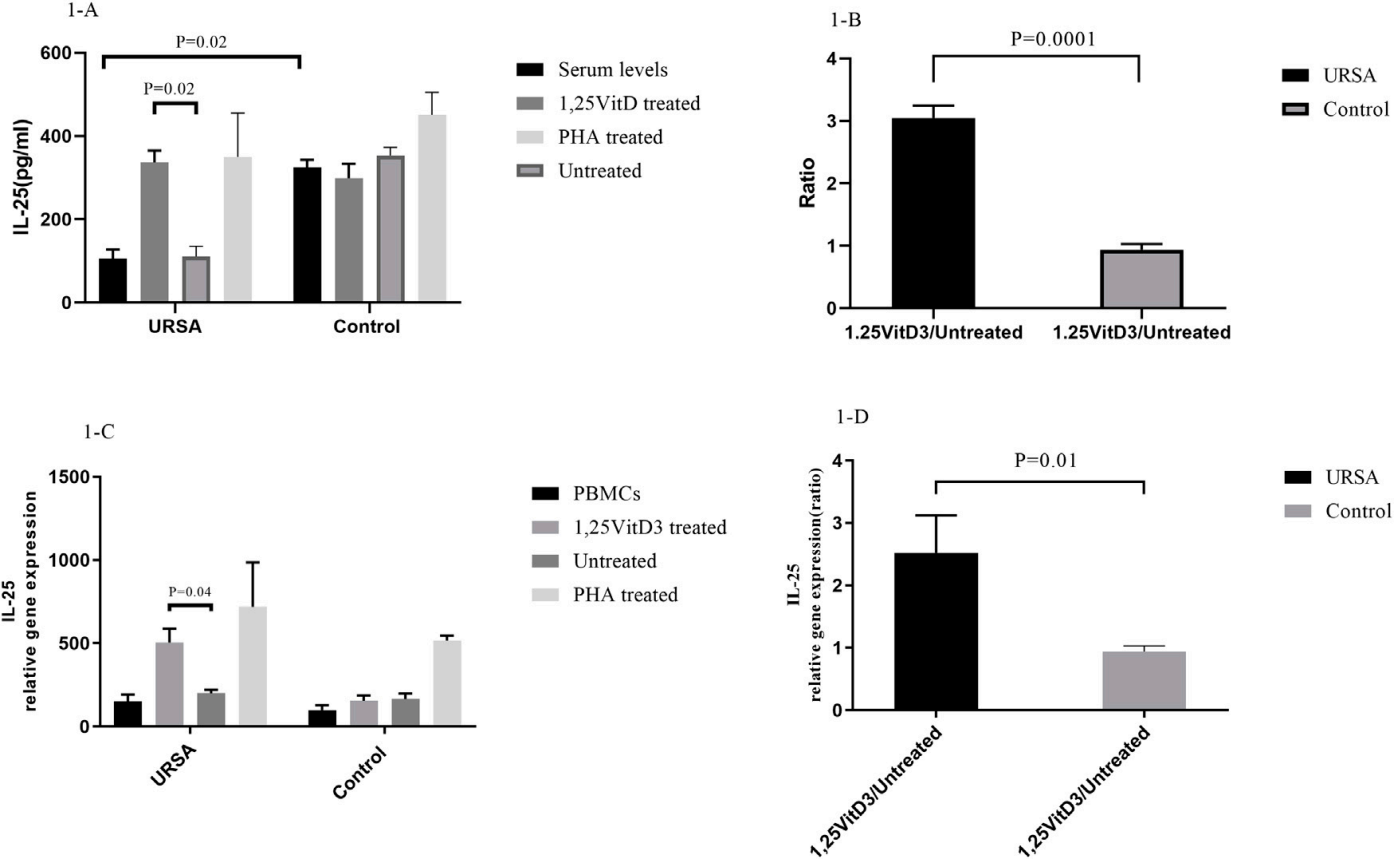
IL-25 expression at mRNA and protein levels. **(A)*.*** IL-25 serum levels were significantly lower in women with URSA than in the control group. IL-25 concentrations in the cell culture supernatants were significantly lower in women with URSA relative to the control group. **(B)**.1,25VitD3/Untreated relative gene expression for IL-25 levels decreased in women with URSA relative to the control group. **(C)**.1,25VitD3 treatment significantly improved IL-25 expression at mRNA level compared to the untreated PBMCs in URSA patients. **(D).** 1,25VitD3 treatment significantly improved IL-25 expression at protein level compared to the untreated PBMCs in URSA patients IL-25 relative gene expression (1,25VitD3/untreated) increased in the case group in comparison to the control group (2.52 ± 0.60 versus. 0.94 ± 0.09; *p* = 0.01; Figure 1D).

IL-25 serum levels were significantly lower in women with URSA than in the control group. IL-25 concentrations in the cell culture supernatants were significantly lower in women with URSA relative to the control group (105.320 ± 22.2 versus. 324.147 ± 18.500, *p* = 0.02; Figure 1A). Furthermore, IL-25 levels (1,25VitD3/Untreated) decreased in women with URSA relative to the control group (3.05 ± 0.20 versus. 0.94 ± 0.09; *p* = 0.0001; Figure 1B).

In the case group, 1,25VitD3 treatment significantly improved IL-25 expression at both levels of mRNA (336.82 ± 28.5 versus. 110.14 ± 25.05; *p* = 0.02; Figure 1C) and protein (336.82 ± 28.51 versus. 110.14 ± 25.05; *p* = 0.02; Figure 1A) compared to the untreated PBMCs.

The relative gene expression of IL-25 (1,25VitD3/untreated) increased in the case group in comparison to the control group (2.52 ± 0.60 versus. 0.94 ± 0.09; *p* = 0.01; Figure 1D).

1,25VitD3 decreased the percentage of Th17 cells in women with RSA.

**FIGURE 2:**
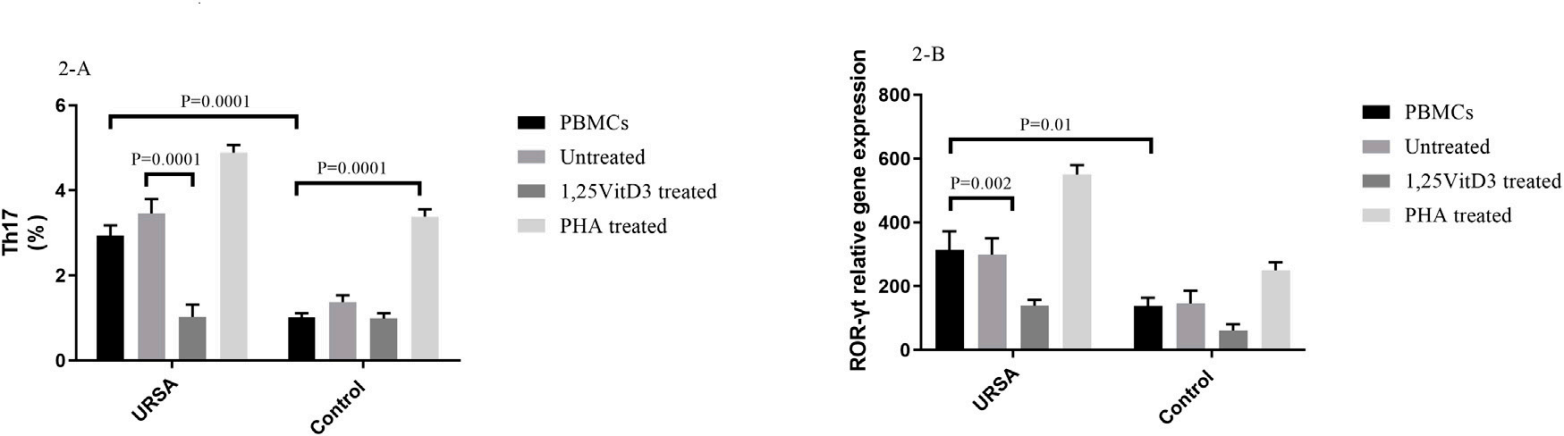
Frequency of Th17 cells and ROR-γt gene expression in URSA and control groups. **(A)**. The proportion of Th17 cells in PBMCs of women with URSA cells were significantly higher compared to the control group. In PBMCs of women with URSA, the percentage of Th17 cells decreased after treatment with 1,25VitD3 relative to untreated PBMCs **(B)**. ROR-γt gene expression was higher in PBMCs of women with URSA than in PBMCs of controls. Treatment with 1,25VitD3 decreased ROR-γt gene expression at the mRNA level relative to untreated PBMCs in women with URSA.

In contrast to PBMCs of the controls, the proportion of Th17 cells in PBMCs of women with URSA cells were significantly higher (2.94 ± 0.24 vs. 1.01 ± 0.09, *p* = 0.0001; Figure 2A). In PBMCs of women with URSA, the percentage of Th17 cells decreased after treatment with 1,25VitD3 relative to untreated PBMCs (1.02 ± 0.28 vs. 3.450 ± 0.34, *p* = 0.0001; Figure 2A), while 1,25VitD3 treatment did not change significantly the frequency of Th17 cells compared to untreated PBMCs in the control group (0.98 ± 0.12 vs. 1.37 ± 0.16, *p* > 0.05; Figure 2A).

1,25VitD3 decreased ROR-γt gene expression in women with URSA.

**FIGURE 3:**
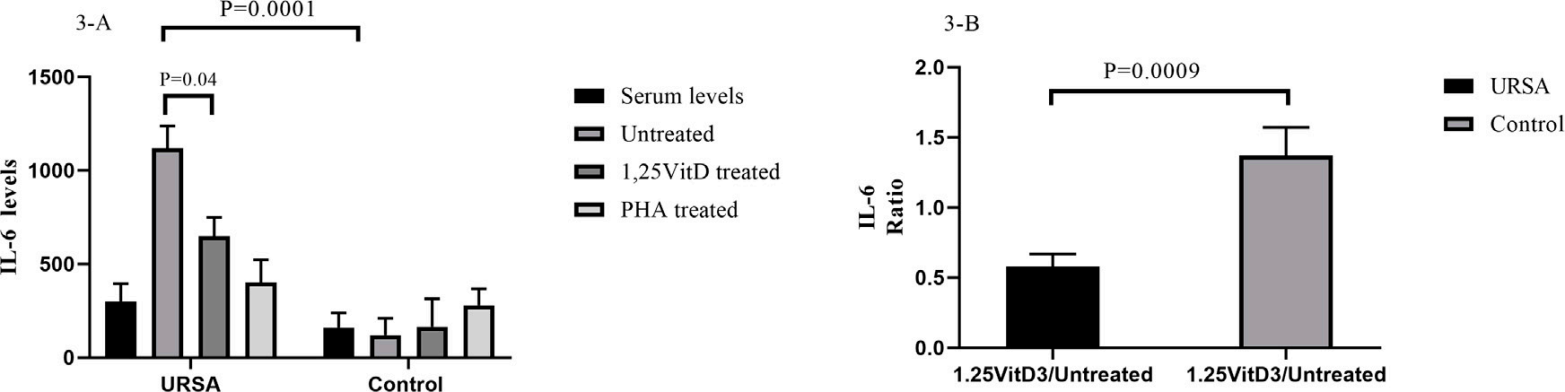
IL-6 levels in serum and cell culture supernatants in women with URSA and the control group **(A)**. IL-6 levels in the cell culture supernatant increased significantly in the case group in comparison to the control group. In cell culture supernatants, 1,25VitD3 treatment decreased IL-6 levels compared to untreated PBMCs only in the case group. **(B)**. IL-6 levels in the presence of 1,25VitD3 to the baseline decreased in women with URSA relative to the control group.

ROR-γt gene expression was higher in PBMCs of women with URSA than in PBMCs of controls (314.24 ± 58.7 vs. 138.14 ± 25.5, *p* = 0.006; Figure 2B). Treatment with 1,25VitD3 decreased ROR-γt gene expression at the mRNA level relative to untreated PBMCs in women with URSA (139.09 ± 50.99 vs. 316.41 ± 50.99; *p* = 0.002; Figure 3B) but not in PBMCs from controls (61.25 ± 19.08 vs. 145.94 ± 40.21; *p* > 0.05; Figure 2B).

1,25VitD3 decreased IL-6 levels in cell culture supernatants in women with URSA relative to the control group.

IL-6 levels in the cell culture supernatant increased significantly in the case group in comparison to the control group (1,120.28 pg/ml ±118.67 versus 1,099.27 pg/ml±118.67; *p* = 0.0001; Figure 3A).

In cell culture supernatants, 1,25VitD3 treatment decreased IL-6 levels compared to untreated PBMCs only in the case group (649.25 pg/ml±99.56 versus 1,120.28 pg/ml ±118.67; *p* = 0.0009; Figure 3A), but not in the control group (*p* > 0.05; Figure 3A). IL-6 levels in the presence of 1,25VitD3 to the baseline decreased in women with RSA relative to the control group (0.63 ± 0.17 versus 0.579 ± 0.09; *p* = 0.0009, Figure 3B).

1,25VitD3 diminished IL-17A levels in women with URSA relative to the control group.

**FIGURE 4:**
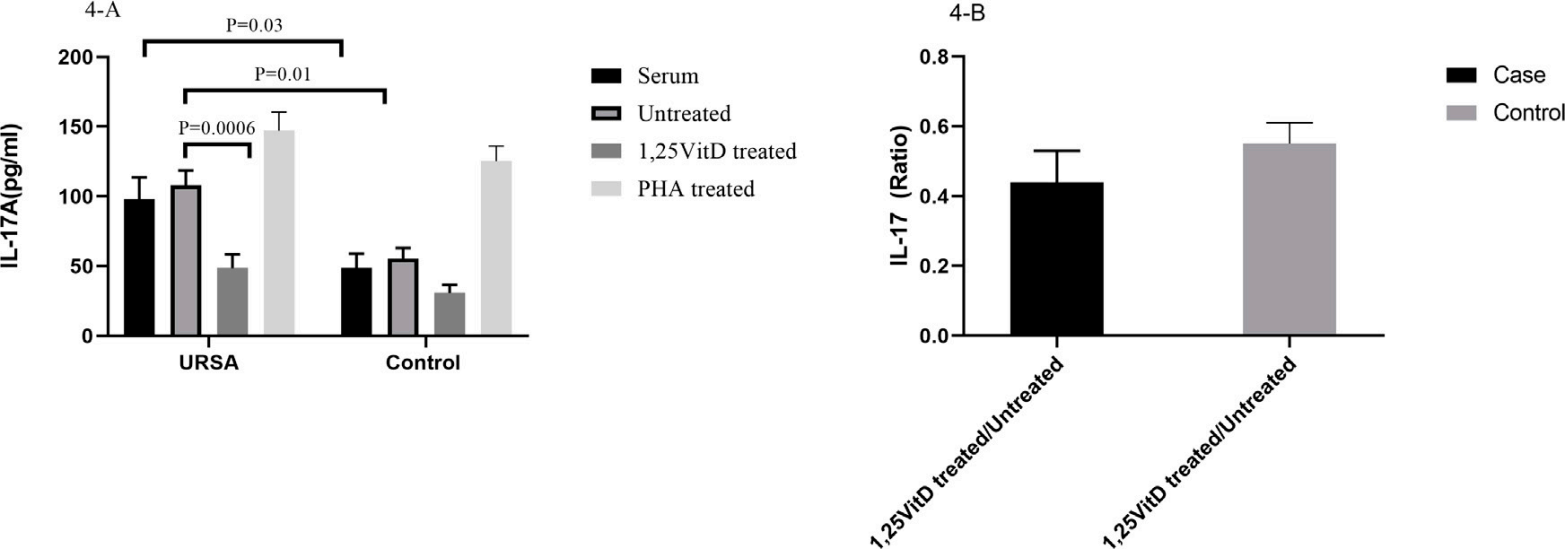
IL-17A levels in serum and cell culture supernatants in women with URSA and the control group **(A)**. IL-17A serum levels significantly increased in the case group in comparison to the control group. IL-17A levels significantly increased in the cell culture supernatants in the case group in comparison to the control group. IL-17A levels decreased in the presence of 1,25VitD3 compared to the absence of that in the case group. **(B)**. IL-17A levels non-significantly decreased in women with URSA relative to the control group.

IL-17A serum levels significantly increased in the case group in comparison to the control group (98.12 ± 15.4 versus 48.5 ± 10.5; *p* = 0.03, Figure 4A). IL-17A levels significantly increased in the cell culture supernatants in the case group in comparison to the control group (108.29 ± 10.4 versus 55.52 ± 7.53; *p* = 0.01, Figure 4A).

IL-17A levels decreased in the presence of 1,25VitD3 compared to the absence of that in the case group (48.6 pg/ml ±10.04 versus 108.29 pg/ml±10.40; *p* = 0.0006; Figure 4A). 1,25VitD3/untreated relative gene expression for IL-17A levels non-significantly decreased in women with URSA relative to the control group (*p* > 0.05, Figure 4B).

1,25VitD3 decreased IL-23 levels in women with URSA relative to the control group.

**FIGURE 5:**
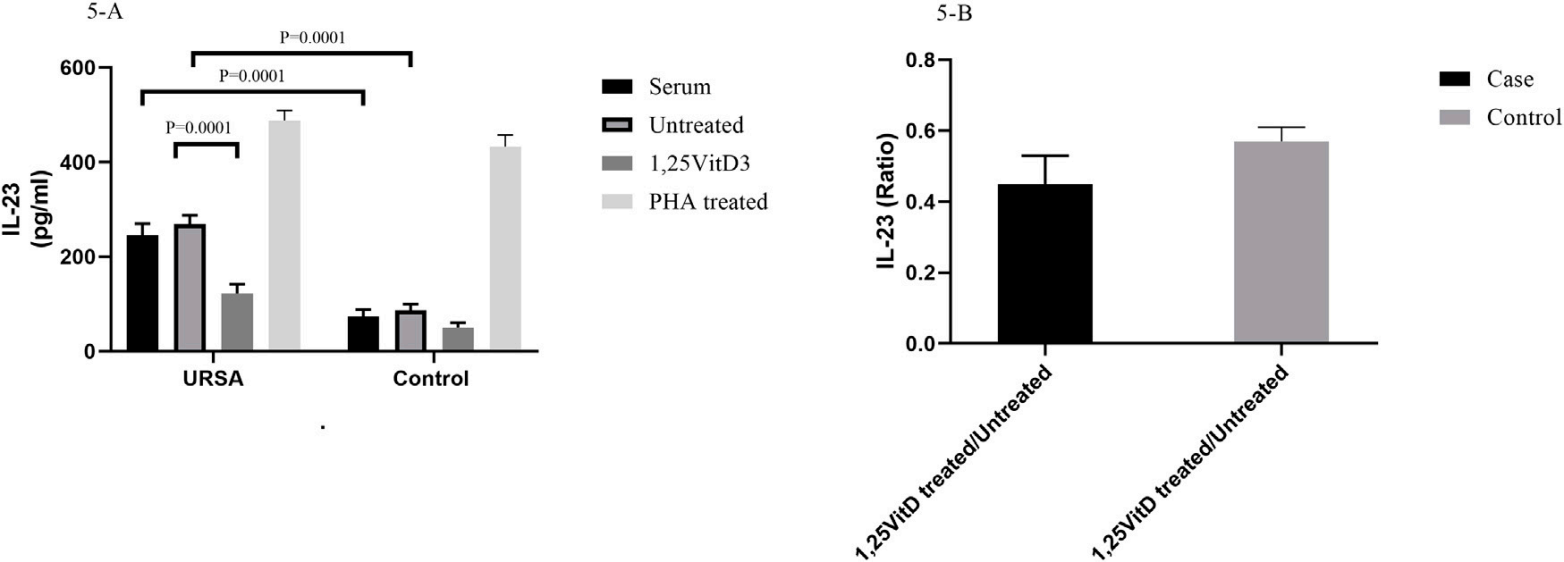
IL-23 levels in serum and cell culture supernatants in women with URSA and the control group **(A)**. IL-23 serum levels were higher in women with RSA than in the control group. IL-23 levels were higher in cell culture supernatants in women with RSA than in the control group. 1,25VitD3 reduced IL-23 levels in the cell culture supernatants in women with URSA in comparison to the control group. **(B)**. IL-123 levels non-significantly decreased in women with URSA relative to the control group.

IL-23 serum levels were higher in women with RSA than in the control group (74/4 pg/ml ± 14/4 versus 245/45 pg/ml± 24/4; *p* = 0.0001; Figure 5A). IL-23 levels were higher in cell culture supernatants in women with RSA than in the control group (268/85 pg/ml ±18/15 versus 87/24 pg/ml±12/86; *p* = 0.0001; Figure 5B).

1,25VitD3 reduced IL-23 levels in the cell culture supernatants in women with URSA in comparison to the control group (89.26 pg/ml±16.47 versus 162.85 pg/ml±19.15; *p* = 0.02; Figure 5B).

## Discussion

Pregnancy is normally considered a state of immunological tolerance, and a break in maternal tolerance may lead to reproductive failure including implantation failure, preeclampsia, preterm birth, and pregnancy loss (Witkin et al., 2011; Ghaneifar et al., 2020). The regulated immune responses are required to protect against harmful pathogens and tolerate a semi-allogeneic fetus expressing paternal antigens in a successful pregnancy (Tsuda et al., 2019). It has been shown that a variety of different immune cells and cytokines maintain maternal immune tolerance to fetal alloantigens during pregnancy (Ali et al., 2020). In a successful pregnancy, a delicate balance has been indicated between various subsets of effector T cells with different secretory cytokines.

In this study, we found that IL-25 levels in serum and supernatants of cell culture were considerably lower in women with URPL than in the healthy women, while IL-17A, IL-23, and IL-6 levels were significantly higher compared to the controls. After PBMCs treatment with 1,25VitD3, IL-25 levels increased, while IL-17A, IL-6, and IL-23 levels decreased in cell culture supernatants in women with URSA relative to the controls. As we previously demonstrated, 1,25VitD3 may reduce the frequency of Th17 cells in PBMCs in women with URPL (Abdollahi et al., 2020b; Abdollahi et al., 2020c). Inflammation has already been identified as the main contributor to inflammatory disorders and pregnancy complications like recurrent spontaneous abortion (Tincati et al., 2009; Pizzola et al., 2016; Karakuş and Çalışkan, 2020). Th17 cells elicit inflammatory reactions by producing IL-17A as the most prominent pro-inflammatory cytokine with a role in URSA occurrence (Veldhoen et al., 2006; Konkel et al., 2017; Fujimoto et al., 2020; Abdollahi et al., 2020d) 63). IL-17A and chemokines including CXCLs attract myeloid cells such as neutrophils to the infection site and activate matrix metalloproteinase, which results in the recruitment of more inflammatory cells such as Th1 and Th17 cells and, as a consequence, a positive loop in amplifying inflammatory reactions (Corrigan et al., 2011; Liu et al., 2016; Abdollahi et al., 2020d).

IL-25 plays an anti-inflammatory role in Th1 and Th17 related disorders, including autoimmune diseases (Selvaraja et al., 2019 Fallon et al., 2006). Similar to the URSA, inflammatory responses of Th17 cells play a key role in the immunopathogenesis of autoimmune disorders such as rheumatoid arthritis (RA), inflammatory bowel disease (IBD), and autoimmune encephalomyelitis. It was found that IL-25 inhibited Th17 cell responses *via* reduction of IL-17A levels and ROR-γt gene expression in PBMCs from patients with RA (Liu et al., 2016; Lavocat et al., 2017). PBMCs are the mixture of mononuclear cells including T cells and monocytes are the appropriate cells for exploring the underlying cellular and molecular mechanisms in immune-mediated diseases including URSA (Ji et al., 19892019; Abdollahi et al., 2015b; Abdollahi et al., 2016b; Abdollahi et al., 2020a; Abdollahi et al., 2020d; Griffiths et al., 2020). Additionally, IL-25 may suppress Th17 cell responses through downregulation of IL-23, IL-1β, and IL-6 expression in activated dendritic cells which may protect mice from severe experimental autoimmune encephalomyelitis (Kleinschek et al., 2007). Furthermore, the deletion of an IL-25-dependent gene resulted in increased Th17 cell function by secreting pro-inflammatory cytokines such as IL-17A leading to exacerbation of CNS disease (Kleinschek et al., 2007).

Of note, IL-25 has been suggested to support successful pregnancy by promoting the proliferation of decidual γδT cells as well as the release of Th2 cytokines like IL-10 (Wang et al., 2012). This resulted in enhancing maternal tolerance (Corrigan et al., 2011; Wang et al., 2014). Decidual γδT cells promote the proliferation and invasion of trophoblast cells as well as suppress the apoptosis *via* IL-10 production (Fan et al., 2011).

IL-25/IL-17RB expression, as the IL-25 receptor, in decidual cells was reported to decrease in women with recurrent abortion compared to normal pregnant women. In early pregnancy, trophoblast-secreted human chorionic gonadotropin (hCG) increased the expression of IL-25 and IL-17RB in decidual stromal cells (Wang et al., 2014; Zhang et al., 2018). This may lead to increased cell proliferation by activating c Jun n terminal kinase (JNK) and protein kinase B (AKT) signals, which could further stimulate DSC proliferation and lead to an increase in the number of DSCs (Wang et al., 2014; Lam et al., 2015).

Additionally, IL-25 may promote human umbilical vein endothelial cell proliferation so it can promote angiogenesis (Corrigan et al., 2011; Wang et al., 2012). In the current study, we indicated that IL-25 levels in the PBMC culture supernatants of the controls were higher than URSA women.

We previously indicated that the active form of VitD3, 1,25VitD3 (50 nm), increased the frequency of Tregs but decreased parentage of Th17 cells at the same dose *in vitro* in women experiencing URSA (Abdollahi et al., 2020d). We also implicated that 1,25VitD3 could increase Treg/Th17 through promoting Treg differentiation and proliferation *via* upregulation of FOXP3 and GITR gene expressions in women with URSA (Ji et al., 2019; Abdollahi et al., 2020c). FOXP3 is a master transcription factor that regulates Treg development and differentiation (van der Veeken et al., 2020). GITR is a marker of the characteristic of Tregs that induces co-stimulatory signals involved in Treg activity (Abdollahi et al., 2020c). As a result, 1,25VitD3 acted as a modulator of the immune system through balancing of Treg/Th17 axis in women with URSA.

Here, we assumed that one of the underlying immunomodulatory mechanisms of 1,25VitD3 may be upregulation of IL-25 expression in PBMCs from patients with URSA. This may inhibit production of Th17 cell inflammatory cytokines including IL-6 and IL-23 in PBMCs from women with URSA.

## Concluding Remarks

Our findings showed that IL-25 expression was lower, while Th17 cell frequency and related cytokines including IL-17A, IL-6, and IL-23 were higher in PBMCs from women with URSA, suggesting that decreasing IL-25 expression in the concordance with increasing inflammatory responses of Th17 cells in PBMCs form women with URPL may involve the immunopathogenesis of URSA. 1,25VitD3 acts as a modulator of the immune system by enhancing the expression of IL-25 that may result in reducing Th17 cells activity. 1,25VitD3enhanced the expression of IL-25 as the anti-inflammatory cytokine. On the other hand, 1,25VitD3 decreased cytokine expressions that were associated with the differentiation or maintenance of Th17 cells (IL-6 and IL-23). Therefore, 1,25VitD3 may decrease inflammatory responses cells *via* down regulating if IL-25 expression in PBMCs from women with URSA. However, more studies with the mechanistic view are warranted to establish this concept. It could be an interesting subject for future clinical trials in the field of the immunopathology of URSA to identify molecular pathways in URSA treatment.

## Data Availability

The datasets presented in this study can be found in online repositories. The names of the repository/repositories and accession number(s) can be found below: https://www.ncbi.nlm.nih.gov/.
